# Perceived School Fairness and Academic Functioning in Early Adolescents: Differential Associations for Adolescents with or Without ADHD?

**DOI:** 10.1007/s10802-025-01419-6

**Published:** 2026-01-30

**Authors:** Andrew C. Martinez, C. Danielle Green, James L. Peugh, Stephen P. Becker

**Affiliations:** 1 Division of Behavioral Medicine and Clinical Psychology, Cincinnati Children’s Hospital Medical Center, 3333 Burnet Avenue, MLC 10006, Cincinnati, OH 45229-3039 USA; 2 Department of Pediatrics, University of Cincinnati College of Medicine, Cincinnati, OH USA

**Keywords:** Adolescence, Attention-deficit/hyperactivity disorder, Learning problems, School climate, Study skills

## Abstract

Although substantial research has focused on the academic outcomes of youth with attention deficit/hyperactivity disorder (ADHD), less is known about factors that promote positive adaptation among these youth in the school setting. Perceived fairness, a subcomponent of school climate, is associated with improved academic functioning and may play an important role among youth with ADHD who have been shown to display heightened reactions to injustice. This study examined perceived fairness in relation to academic functioning in early adolescents and whether this association differed for adolescents with or without ADHD. Participants were 341 early adolescents (ages 10–12), with approximately half (48.7%) diagnosed with ADHD. Teachers completed measures of learning problems and study skills, and adolescents completed measures assessing perceived fairness and attitude to school. Students with ADHD perceived their school to be less fair than students without ADHD, though the effect size was small. Regression analyses controlling for adolescent medication use, demographic characteristics, and co-occurring psychopathology revealed significant main effects wherein higher levels of perceived fairness predicted lower negative attitude to school, and ADHD status predicted a more negative attitude to school and lower study skills. A significant perceived fairness × ADHD interaction was found in relation to learning problems, wherein higher perceived school fairness was more strongly associated with fewer learning problems for students with ADHD than for students without ADHD. These findings contribute to research exploring promotive factors among adolescents with and without ADHD and have implications for interventions aiming to improve academic functioning.

Attention-deficit/hyperactivity disorder (ADHD) is characterized by a developmentally inappropriate pattern of inattention and/or hyperactivity-impulsivity and is broadly associated with poorer educational and socio-emotional functioning (Froehlich & Becker, 2025). Substantial research has focused on academic functioning, a domain in which youth with ADHD face a particularly high risk of negative outcomes (DuPaul & Jimerson, 2014). As a group, youth with ADHD exhibit lower academic achievement, obtain lower grades, and are at greater risk for grade retention and expulsion, compared to their peers without ADHD (DuPaul & Langberg, 2015; Loe & Feldman, 2007). Further, students with ADHD display interpersonal challenges, having poorer relationships with peers and teachers than students without ADHD (Ewe, 2019; Gardner & Gerdes, 2015). Despite these well-established associations between ADHD and a broad range of functional impairments within the school setting, outcomes for children with ADHD are heterogenous, with some children functioning as well as or better than their peers (Dvorsky & Langberg, 2016; Sonuga-Barke et al., 2023). Given this, there is growing interest in considering factors that may promote positive adaptation among youth with ADHD, moving beyond a primary focus on risk factors and deficits (Dvorsky & Becker, 2025). In addition, as the vast majority of ADHD-focused research has emphasized individual-level factors (e.g., organization, social skills, psychiatric comorbidity), there is a recognized need to also examine environmental factors that may be promotive for youth with ADHD, in line with calls to examine resilience at multiple levels (Wright & Masten, 2015). Accordingly, the current study focuses on school climate as an environmental factor that may be particularly promotive for youth with ADHD.

## School Climate

School climate refers to patterns of people’s experiences in school which reflect norms, goals, values, interpersonal relationships, teaching and learning practices, and organizational structures (Thapa et al., 2013). Broadly, positive perceptions of school climate are consistently associated with positive outcomes including greater self-esteem and higher academic achievement (Jia et al., 2009; Joyce & Early, 2014; Kidger et al., 2012). Conversely, negative perceptions of school climate have been associated with negative outcomes such as poorer self-esteem, depressive symptoms, and increased problem behavior (Way et al., 2007). Much of the research examining correlates of school climate has focused on internalizing psychopathology (Kidger et al., 2012), with far fewer studies examining associations to other domains such as educational outcomes (Cohen et al., 2009). If school climate is associated with educational outcomes, particularly for students with ADHD who are at increased risk for negative academic functioning, this may help build more comprehensive conceptual models and open avenues for optimizing interventions to promote both school climate and academic success.

## ADHD and School Climate

Surprisingly few studies have examined ADHD in the context of school climate, with mixed findings reported to date. In a small study exploring the lived experience of students with ADHD (*N* = 20, ages 12–14), having close friendships at school and the warmth and openness of their teachers were identified as key factors that contribute to successful school functioning (Krtkova et al., 2023). In a larger sample of school-aged children (*N* = 4,114, ages 6–18), Chan et al. (2018) found that children with disabilities, including ADHD, were more likely to experience victimization in typical school settings compared to special schools which are designed to serve children with unique educational needs or challenges (e.g., Intellectual Disability). More recently, in a longitudinal study using a sample of 288 early adolescents (ages 13–15) with and without ADHD, higher perceived school support was directly related to decreased depressive symptoms, increased academic competence, and increased self-efficacy (Green et al., 2023). However, these associations were not moderated by ADHD status, suggesting that the school climate may be similarly meaningful for students with and without ADHD. These limited and inconsistent findings point to the need for additional studies and raise the possibility that specific subcomponents of school climate may provide a more nuanced understanding of the role of school climate for youth with ADHD.

## Perceived School Fairness

One specific yet less-studied aspect of school climate that may be of particular importance is perceived fairness. Although defined in somewhat differing ways across studies (Rasooli et al., 2023), perceived fairness as defined in this study refers to a student’s perception about how all students are treated in their school. Studies in general samples of students have shown that positive perceptions of fairness are associated with positive outcomes in the school setting. In a recent review, Chory (2023) found that grading procedures in addition to instructor feedback, affect, attention, and caring were key factors in determining levels of “classroom justice,” which is itself associated with student learning, engagement, and antisocial classroom behavior at the university level. Negative perceptions of fairness have been shown to predict higher levels of perceived alienation amongst university students in a Turkish sample (Çağlar, 2013). More recently, Wong et al. (2022) found that, in a sample of 269 high school students, more positive perceptions of teacher fairness were associated with higher ratings of school belonging, a factor that has a robust impact on overall school functioning (Slaten et al., 2016).

Perceived school fairness may not just impact feelings about school, but also how students engage, behave, and perform at school. When students perceive the school environment as fair, they may be more likely to engage with learning tasks and internalize academic expectations. This can foster greater motivation and self-regulation, which are key to the development of effective study skills and reduced learning problems (Villar et al., 2024; Zimmerman, 2002). Conversely, perceived unfairness may exacerbate disengagement or oppositional behavior, interfering with academic functioning. In this vein, Chan et al. (2025) found that in a longitudinal study of 274 adolescents (140 with ADHD; 8th grade at timepoint 1, 10th grade at timepoint 2), higher ratings of disciplinary structure, defined as a strict but *fair* environment, promoted increased homework performance and academic motivation, providing support that fairness related constructs may be uniquely important to overall academic functioning. Although more is known about the role of perceived fairness for older adolescents and emerging adults (Çağlar, 2013; Chory, 2023; Wong et al., 2022), it is important to further understand this construct in early adolescence. In particular, early adolescence is a developmental period characterized by major biopsychosocial changes (e.g., interpersonal relationships, identity formation, pubertal development; Archibald et al., 2006; Kenny et al.,^2013^; Kroger et al., 2010), which can be a sensitive time for shaping perceptions of fairness. It may be the case that early experiences and perceptions of unfairness in schools create a negative attitude to the way students are treated in schools that is difficult to improve and remains stable over time.

To our knowledge, Chan et al. (2025) are the first to also examine the specific role of a fairness-related construct in relation to academic functioning and ADHD symptom severity. Perceived fairness may play an especially important role for youth with ADHD given that individuals with ADHD display increased justice sensitivity (Bondü & Esser, 2015; Schäfer & Kraneburg, 2015). Justice sensitivity is a well-established construct that describes individual differences in the frequency in which injustice is perceived and the intensity of emotional, cognitive, and behavioral reactions to injustice (Baumert et al., 2022). Research suggests that individuals displaying increased justice sensitivity are more likely to perceive injustice and react more strongly when they encounter an unjust situation. Theoretical models propose that frequent experiences of injustice may lead to an increased activation potential of injustice-related concepts, which in turn could lead to increases in justice sensitivity across time (Baumert & Schmitt, 2009). Empirical research has supported this theory, finding that being confronted with injustice increases state levels of self-reported justice sensitivity (Wijn & van den Bos, 2010) and that across 6-months, undergraduate students who reported more frequent injustice showed relative increases to ratings of justice sensitivity compared to their peers who reported less frequent injustices (Baumert & Maltese, 2014). Thus, it has been theorized that youth with ADHD may be more likely to develop heightened justice sensitivity as a result of facing frequent injustice as a by-product of their ADHD related challenges (Bondü & Esser, 2015; Schäfer & Kraneburg, 2015). Consequently, youth with ADHD may be more likely to perceive injustice and may react more intensely when injustice is perceived and therefore may be at risk to develop negative perceptions toward the fairness of their school environment. Considering the relationship between perceptions of fairness and academic functioning, as detailed above, the relationship between perceived fairness and academic functioning may be particularly relevant for adolescents with ADHD.

## Present Study

The present study sought to examine perceived school climate fairness in relation to academic functioning in a sample of 341 adolescents with and without ADHD. Using a multi-informant approach, ADHD status was examined as a moderator of the association between adolescent-rated perceptions of school climate and attitudes to school and teacher-rated study skills and learning problems. The aims of the current study are to (1) compare the perceptions of fairness in youth with and without ADHD, (2) examine the relationship between perceptions of fairness and academic functioning, and (3) test ADHD status as a potential moderator of the association between perceived fairness and academic functioning. Considering the literature highlighted above, we hypothesized that adolescents with ADHD will have more negative perceptions of school climate fairness than adolescents without ADHD. Additionally, we hypothesized that more positive perceptions of perceived school fairness would be associated with more positive attitudes toward school, higher study skills, and lower learning problems. Lastly, we expected ADHD status to serve as a moderator between perceived fairness and each academic functioning outcome, with the effects being stronger or present for youth with ADHD more so than for youth without ADHD.

## Methods

### Participants

Participants were 341 early adolescents (ages 10–12 years) recruited from 214 different schools. Characteristics of the adolescent participants and their caregiver/family are summarized in Table1. There were approximately an equal number of female and male adolescents. Approximately half of the sample met diagnostic criteria for ADHD based on the Kiddie Schedule for Affective Disorders and Schizophrenia for School-Age Children (K-SADS; Kaufman et al., 2013) conducted with the adolescent’s caregiver (primarily biological mothers).

**Table 1:** Sample characteristics (*N* = 341)

Adolescent Characteristics		Caregiver/Family Characteristics	
	*M* ± *SD*		*n* (%)
Age	10.90 ± 0.80	Relationship to Child	
		Biological Mother	293 (85.9)
	*n* (%)	Biological Father	26 (7.6)
Sex		Stepmother	2 (0.6)
Female	178 (52.2)	Adoptive Mother	14 (4.1)
Male	163 (47.8)	Adoptive Father	1 (0.3)
		Foster Mother	1 (0.3)
Race		Grandmother	3 (0.9)
American Indian/Alaskan	1 (0.3)	Grandfather	1 (0.3)
Asian	8 (2.3)		
Black	72 (21.1)	Household Income^b^	
Multiracial	48 (14.1)	Under $20,000	15 (4.5)
White	212 (62.2)	20,001–40,000	37 (11.1)
		40,001–60,000	34 (10.2)
Hispanic/Latinx	32 (9.4)	60,001–80,000	38 (11.4)
		80,001–100,000	34 (10.2)
Psychiatric diagnoses^a^		100,001–120,000	45 (13.5)
ADHD - PR	166 (48.7)	Over $120,000	130 (39.0)
Any externalizing (ODD)^a^ - PR	24 (7.0)		
Any anxiety - PR	52 (15.2)	Highest Primary Caregiver Education^c^	
Any anxiety - SR	39 (11.4)	High school degree or less	25 (7.4)
Any depression - PR	5 (1.5)	Partial college/vocational	51 (15.1)
Any depression - SR	8 (2.3)	College graduate	141 (41.7)
		Graduate/professional degree	121 (35.8)
Medication Use			
ADHD	83 (24.3)		
Other psychiatric	24 (7.0)		

Note. ADHD = attention-deficit/hyperactivity disorder. ODD = oppositional defiant disorder. Anxiety disorders = presence of generalized anxiety disorder, social phobia, panic disorder, and/or posttraumatic stress disorder (PTSD). Any depression = presence of major depression or dysthymia PR = parent-report. SR = adolescent self-report

^a^ Current psychiatric diagnoses were assessed using the Kiddie Schedule for Affective Disorders and Schizophrenia for School-Age Children (K-SADS). Only caregivers were administered the ODD and PTSD modules. No participants met criteria for conduct disorder

^b^ Eight caregivers did not provide family income

^c^ Three caregivers did not provide education

Inclusion criteria also included a standardized score ≥ 80 on the Peabody Picture Vocabulary Test, 5th Edition (Dunn, 2019), willingness to discontinue stimulant medications for ADHD 24 h prior to their research visit, and sufficient English language ability to complete the measures. In addition, children were excluded if the caregiver reported a previous diagnosis of autism spectrum disorder, bipolar disorder, or psychosis, or a significant visual, hearing, or speech impairment precluding their ability to complete the measures.

## Procedures

All procedures were approved by the Cincinnati Children’s Hospital Medical Center Institutional Review Board. Participants were recruited from a variety of sources, including media advertisements, community flyers, e-mail distribution within a Midwestern children’s hospital, and letters to school and pediatrician partners. To ensure the full range of attentional symptoms, several versions of advertising materials were used (e.g., with and without descriptors of attentional problems). For additional details, see Becker et al. (2024).

Interested caregivers completed a brief REDCap survey that included initial inclusion criteria (e.g., child’s age). Families meeting initial inclusion criteria were scheduled for an in-person research visit. Given the study visit included the collection of physiological data during a task (unrelated to the present study), participants taking stimulant medication for ADHD were instructed to withhold medication on the day of their research visit. After caregivers and adolescents provided informed consent and assent, respectively, study measures were collected. Caregivers also signed a release to gather teacher ratings, which were obtained for 256 of the 341 participants included in this study. Participants with and without teacher data did not significantly differ in ADHD status, current medication status, age, sex, race, ethnicity, school fairness, or adolescent self-reported attitude to school (all *p*s > 0.05). Participants without teacher ratings had lower family income than participants with teacher ratings (*p* =.021).

## Measures

Caregivers completed a demographic form to gather the information reported in Table 1. Medication use and psychosocial treatment was assessed with an adaptation of the Services Use in Children and Adolescents - Parent Interview (SCA-PI; Eaton Hoagwood et al., 2004).

### Kiddie Schedule for Affective Disorders and Schizophrenia for School-Age Children (K-SADS)

The K-SADS (Kaufman et al., 2013) is a semi-structured diagnostic interview based on the DSM-5 with good reliability and validity (Jarbin et al., 2017). The ADHD, conduct disorder (CD), generalized anxiety disorder, social phobia, panic disorder, post-traumatic stress disorder, major depression, and persistent depressive disorder modules of the K-SADS were administered to parents and adolescents separately. The oppositional defiant disorder (ODD) module was administered to parents only. Rating scale data support the concurrent validity of screens and K-SADS diagnoses (Kaufman et al., 1997). Inter-rater agreement in scoring screens and diagnoses is high (range: 93% to 100%) (Kaufman et al., 1997). The K-SADS was administered by trained graduate students, post-doctoral fellows, or clinical psychologists to caregivers and adolescents separately. All interviewers underwent a standardized training process, including an overview and in-depth training on the K-SADS structure and individual modules/items, scoring alongside previously recorded interviews, meeting to review any discrepancies with an experienced licensed psychologist, observing live interviews, and being observed conducting live interviews before conducting interviews independently. Throughout each training year, a handful of K-SADS interviews were recorded and coded for reliability, which was 100%.

### School Climate Survey, Elementary and Middle School Version (SCS)

The School Climate Survey (Haynes et al., 1997) was completed by adolescents to assess their perceptions of fairness in their schools. The SCS is a well validated measure used to assess students’ perceptions of their school climate, including a 5-item subscale focused on fairness (α = 0.78). Fairness items assess how students are treated at school (e.g., “everyone is treated equally well at my school,” “at my school, children of all races are treated the same”).

### Behavioral Assessment System for Children, Third Edition (BASC-3)

The BASC-3 (Reynolds & Kamphaus, 2015) was completed by adolescents and their teachers to assess academic functioning. The BASC-3 is a reliable and well-validated multidimensional measure to evaluate emotional and behavioral functioning in children. Attitude to school assesses negative attitudes toward school using the self-report scale (eight items rated as *true* or *false* or on a 4-point scale [1 = *never*, 2 = *sometimes*, 3 = *often*, 4 = *almost always*]; e.g., “I don’t care about school,” “I hate school”). The teacher-report version includes scales assessing learning problems and study skills, with all items rated on a four-point scale (see above). The learning problems subscale contains 8 items (e.g., “performs poorly on school assignments,” “has problems with mathematics”). The study skills subscale contains 8 items for children younger than 12 (child version) (e.g., “turns in work on time”) and 11 items for children 12–21 (adolescent version) (e.g., “has good study habits”). For BASC-3 subscales, *T*-scores were used, with higher attitudes towards school scores indicating a more negative attitude toward school, higher learning problems scores indicating greater learning problems, and higher study skills scores indicating better study skills.

### Analytic Approach

First, IBM SPSS Statistics Version 29 statistical software was used to conduct zero-order correlations among study variables. A correlation of 0.10 is generally considered a small effect, 0.30 is considered a medium effect, and 0.50 is considered a large effect. Primary analyses were conducted in *BLIMP* (version 3.2.20; Keller & Enders, 2023). Multiple imputation is recommended for missing data handling if the analysis involves moderation (Enders et al., 2020). *BLIMP* allows for model-based multiple imputation (Peugh & Mara, 2024) and analysis model parameter estimation in a single step. Any nesting within schools was also handled appropriately using *BLIMP*, including handling clusters with only one member (i.e., “singleton clusters” in which a single student from a single school included in the sample) which constitutes a partially nested design (Keller & Enders, 2023). Perceived school fairness and ADHD group diagnostic status, as well as their interaction, were the primary independent variables predicting negative attitude to school, study skills, and learning problems. As previous research has shown medication use, demographic factors, and co-occurring psychiatric conditions to be related to academic functioning (Langberg & Becker, 2012; Battle & Lewis, 2002; Pagerols et al., 2022) as well as in our sample (see Table 2), medication use (binary indicator), age, race/ethnicity (binary indicator), income, and both internalizing and externalizing psychological disorders (binary indicators) were included as covariates in the current study^1^. A more rigorous potential scale reduction factor (PSR < 0.01) was used to ensure both the convergence and stability of the model-based multiple imputation. Simple slope analyses were used to follow-up any significant interaction effects.

**Table 2:** Intercorrelations and descriptive statistics of study variables

Variable	1	2	3	4	5	6	7	8	9	10	11	12	13
1. Medication status	--												
2. Age	0.03	--											
3. Sex	− 0.15**	0.06	--										
4. Race	0.14**	0.10	− 0.02	--									
5. Ethnicity	0.004	− 0.01	0.07	0.04	--								
6. Family income	0.07	0.05	− 0.07	0.26***	0.01	--							
7. ADHD status	0.49***	− 0.01	− 0.18***	0.09	− 0.07	− 0.06	--						
8. Ext. disorder	0.16**	0.02	− 0.01	0.03	0.07	0.000	0.17**	--					
9. Int. disorder	0.20***	0.05	0.08	0.12*	0.03	0.04	0.22***	0.11*	--				
10. School fairness	− 0.16**	− 0.04	− 0.002	0.07	− 0.06	0.02	− 0.11*	− 0.08	− 0.17**	--			
11. Attitude to school	0.17**	− 0.07	− 0.09	− 0.08	− 0.002	− 0.04	0.20**	0.16**	0.06	− 0.42***	--		
12. Study skills	− 0.18**	0.13*	0.22***	0.13*	0.07	0.24***	− 0.39***	− 0.17**	0.04	0.23***	0.34***	--	
13. Learning problems	0.19**	− 0.08	− 0.19**	− 0.06	− 0.12	− 0.24***	0.39***	0.08	0.03	− 0.29***	0.27***	− 0.80***	--
*M*	--	10.90	--	--	--	--	--	--	--	2.68	53.90	47.80	50.94
*SD*	--	0.80	--	--	--	--	--	--	--	0.44	11.26	10.79	10.25

*Note. N =* 341 (*n =* 256 for teacher-rated variables). For medication status, 0 = not currently taking any prescribed psychiatric medication, 1 = currently taking a prescribed psychiatric medication. For sex, 0 = male, 1 = female. For race, 0 = person of color, 1 = White. For ethnicity, 0 = non-Hispanic, 1 = Hispanic. For ADHD, 0 = non-ADHD, 1 = ADHD. ADHD = attention-deficit/hyperactivity disorder

*p <.05. **p <.01. ***p <.001

## Results

### Bivariate Correlations

Table 2 provides the intercorrelations and descriptive statistics of the study variables. In considering our primary variables, compared to adolescents without ADHD, adolescents with ADHD reported lower perceived school fairness (*p* =.046) and having a more negative attitude to school (*p* <.001). Teachers also rated adolescents with ADHD as having poorer study skills (*p* <.001) and higher learning problems (*p* <.001) than adolescents without ADHD. Lower perceived school fairness was associated with a more negative attitude to school (*p* <.001), poorer study skills (*p* <.001), and higher learning problems (*p* <.001). Finally, a more negative attitude to school was associated with lower teacher-reported study skills (*p* <.001) and higher teacher-reported learning problems (*p* <.001).

### Regression Analyses Examining ADHD as a Moderator of Perceived School Fairness and Academic Outcomes

Results of the regression analyses examining perceived school fairness, ADHD group status, and their interaction in relation to academic outcomes are summarized in Table 3.

**Table 3:** School fairness in relation to academic functioning and moderation by ADHD status

	Standardized Estimate (SD)	X^2^	95% CI
*Attitude to School*	*R*^2^ = 0.23		
Age	−0.07 (0.05)	2.00	−0.16, 0.03
Race	−0.05 (0.05)	0.94	−0.15, 0.05
Medication use	0.05 (0.06)	0.77	−0.06, 0.16
Family income	−0.02 (0.05)	0.09	−0.11, 0.08
Externalizing disorder	0.10 (0.05)	4.30*	0.01, 0.19
Internalizing disorder	−0.05 (0.05)	0.95	−0.15, 0.05
School Fairness	−0.46 (0.07)	46.04***	−0.58, −0.32
ADHD Status	0.13 (0.06)	5.44*	0.02, 0.24
Fairness × ADHD	0.10 (0.07)	1.88	−0.04, 0.24
*Study Skills*	*R*^2^ = 0.28		
Age	0.14 (0.05)	6.89**	0.03, 0.24
Race	0.12 (0.06)	4.41*	0.01, 0.22
Medication use	0.02 (0.06)	0.13	−0.10, 0.14
Family income	0.11 (0.06)	3.39^*^	−0.01, 0.22
Externalizing disorder	−0.09 (0.05)	3.21^†^	−0.19, 0.01
Internalizing disorder	0.11 (0.06)	4.06*	0.00, 0.22
School Fairness	0.08 (0.09)	0.81	−0.10, 0.26
ADHD Status	−0.37 (0.06)	39.03***	−0.48, −0.25
Fairness × ADHD	0.15 (0.09)	2.86	−0.02, 0.31
*Learning Problems*	*R*^2^ = 0.27		
Age	−0.10 (0.05)	3.12	−0.20, 0.01
Race	−0.05 (0.06)	0.88	−0.16, 0.06
Medication use	−0.02 (0.06)	0.11^†^	−0.06, −0.14
Family income	−0.13 (0.06)	5.17^*^	−0.24, −0.02
Externalizing disorder	−0.01 (0.05)	0.07	−0.12, 0.09
Internalizing disorder	−0.04 (0.06)	0.58	−0.15, 0.07
School Fairness	−0.12 (0.09)	1.74	−0.30, 0.06
ADHD Status	0.36 (0.06)	34.77***	0.23, 0.47
Fairness × ADHD	−0.19 (0.09)	4.63*	−0.35, −0.01

Note. Bootstrap samples = 5,000. For medication status, 0 = not currently taking any prescribed psychiatric medication, 1 = currently taking a prescribed psychiatric medication. For race, 0 = person of color, 1 = White. For externalizing disorder, 1 = presence of an externalizing disorder. For externalizing disorder, 1 = presence of an internalizing disorder. ADHD = attention-deficit/hyperactivity disorder

^†^*p* <.10. ^‡^*p* =.06. *p <.07. **p <.01. ***p <.001

In analyses controlling for participant characteristics, family income, co-occurring disorders, and medication status, significant main effects were observed for perceived fairness and ADHD diagnostic status predicting negative self-reported attitudes toward school. Higher levels of perceived fairness were associated with significantly lower negative attitudes toward school (estimate = −0.46, *p* <.001), whereas an ADHD diagnosis significantly predicted higher negative school attitudes (estimate = 0.13, *p* =.020). The perceived fairness × ADHD group interaction was not significant (estimate = 0.10, *p* =.170). In considering study skills, an ADHD diagnosis significantly predicted lower teacher-reported study skills (estimate = −0.37, *p* <.001) as a main effect not moderated by fairness perceptions.

**Fig. 1:**
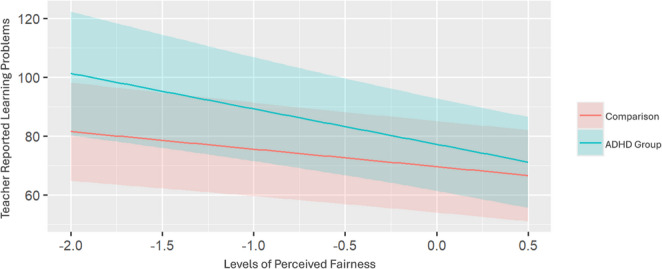
ADHD Status Moderates the Association between School Fairness and Teacher-Reported Learning Problems. Note.The line with the stronger slope (top line) is the ADHD group; the line with the weaker slope (bottom line) is the comparison group. ADHD=attention-deficit/hyperactivity disorder

The perceived fairness × ADHD interaction was significant in the model predicting learning problems (estimate = −0.19, *p* =.031; see Table 3). As shown in Fig. 1, above and beyond covariates, simple slope conditional effect analyses indicated that higher perceived school fairness was more strongly associated with lower learning problems for students with ADHD (coefficient = −12.06, *p <*.001) than for students without ADHD (coefficient = *-*5.95, *p* = < 0.001). Additionally, regions of significance calculations were conducted and revealed that the moderating effect of ADHD status is no longer significant at moderate to high levels of perceived fairness (< −1.5 standard deviations below the mean).

## Discussion

The purpose of this study was to examine associations between perceived fairness and academic functioning among early adolescents with and without ADHD. Consistent with our hypotheses, students with ADHD perceived their school climate to be less fair than students without ADHD, though this effect size was small. In addition, ADHD status was significantly associated with more negative attitudes to school, lower study skills, and higher learning problems. In contrast to our expectations, higher perceived fairness was only found to predict lower negative attitudes to school in the full sample. Regarding our moderation analyses, a significant ADHD × perceived fairness interaction was only found for learning problems, such that higher ratings of perceived fairness were more strongly associated with lower ratings of learning problems for students with ADHD than for students without ADHD.

Considering the well-established links between school functioning challenges among youth with ADHD, the observed group difference in perceived fairness is unsurprising. Youth with ADHD often experience more disciplinary actions, receive less positive attention from teachers, and experience greater peer rejections, all of which can contribute to a heightened perception of students being treated unfairly at school (Arnold et al., 2020; McQuade & Hoza, 2015; Ewe, 2019). Our findings serve as further evidence of the inherent challenges faced by youth with ADHD in the academic setting. However, our results concerning perceived fairness-related main effects, namely that perceptions of fairness were not associated with higher study skills or lower learning problems, are somewhat surprising, considering the robust associations between school climate and broad academic functioning (Cohen, 2009). However, most studies on school climate and academic functioning have not included a specific focus on perceived fairness, which might relate more narrowly to specific aspects of academic functioning such as attitude to school. Moreover, other facets of school climate (e.g., student-teacher relations, parental involvement, school safety) may more directly relate than fairness perceptions specifically to academic functioning.

To our knowledge, this is the first study examining the moderating effect of ADHD status on the association between perceptions of school fairness and academic functioning. Our findings suggest that perceived fairness may play a unique role in the academic functioning of adolescents with ADHD, relative to their peers without ADHD. The observed association between higher levels of perceived fairness and lower levels of teacher-reported learning problems being stronger for youth with ADHD compared to youth without ADHD is consistent with the limited body of research linking heightened justice sensitivity to ADHD (Bondü & Esser, 2015; Schäfer & Kraneburg, 2015). It may be the case that students with ADHD are more likely to identify and perceive situations (e.g., grading policies or rule enforcement) as unfair or unjust and may also in turn respond more negatively to them. In contrast, youth without ADHD may be less reactive to such perceived injustices or less likely to interpret them as unfair in the first place. Over time, the more frequent and intense responses to perceived injustice in the school setting could lead to worse perceptions of school climate fairness, which is associated with a host of negative outcomes and academic risk factors such as poorer engagement, belongingness, and increased alienation (Chory, 2023; Wong et al., 2022; Çağlar, 2013). Conversely, when students perceive their school environment as fair and just, they may feel a greater sense of belonging and trust in their teachers, which could promote engagement, motivation, and in turn fewer learning problems.

Our results suggest that perceptions of fairness may serve as a promotive factor particularly for early adolescents with ADHD that could help ameliorate the observed gap in overall learning problems. The regions of significance calculations suggest that higher levels of perceived fairness reduce the discrepancy in learning problems between early adolescents with and without ADHD, such that they are no longer significantly different at moderate to high levels of perceived fairness. These findings underscore the potential of perceived school fairness as a modifiable context that may buffer against academic difficulties for adolescents with ADHD.

In contrast to our findings for learning problems, we did not find a significant ADHD × perceived fairness interaction for attitude to school or study skills. There are a few possible explanations for the lack of moderation related to these outcomes. First, it is important to note that attitude to school is a relatively broad construct compared to learning problems. Given the wide range of impairments and risk factors experienced by youth with ADHD in the school setting (DuPaul & Langberg, 2015), many factors may contribute to negative school attitudes. It could be that the multitude of school problems faced by adolescents with ADHD, such as poor peer and teacher relationships (Ewe, 2019; McQuade & Hoza, 2015) or poor overall academic achievement (Arnold et al., 2020), blur the impact that perceptions of fairness may have on one’s overall attitude to school. That is, even if adolescents with ADHD view their school environment to be fair, other challenges may still negatively affect their attitudes toward school.

Additionally, ADHD-related executive functioning (EF) deficits may lead to persistent problems with study skills regardless of the perceived fairness of the school climate. Although EF deficits can also contribute to learning problems, learning problems may be more context-sensitive and more directly influenced by students’ immediate emotional or behavioral responses to perceived unfairness. For instance, if a student feels they are being treated unfairly, they may disengage and resist academic demands, which can interfere with task completion and focus. These reactive behaviors are more likely to manifest as learning problems than as shifts in broader attitudes or academic skills. Additional research will be needed to more directly test these possibilities.

Despite having controlled for co-occurring disorders in the analyses, it is also important to consider how different types of psychopathologies may impact perceptions of fairness and how they relate to academic functioning. For example, an individual with an externalizing disorder such as oppositional defiant disorder (ODD) may be more likely to feel resentful or irritable in the school setting and defy school rules more frequently. Together, these traits could lead individuals with ODD to be even more likely to view their school as unfair and have even stronger links to academic deficits. On the other hand, an individual with an internalizing disorder might display increased fear and worry associated with school performance, a potential component of generalized anxiety disorder, or display a heightened sensitivity to perceived threats in the school system such as school victimization, a potential post-traumatic stress response (McLoon et al., 2006; Kimble et al., 2014). As a result, these individuals may experience or perceive fairness in a unique, potentially more sensitive way compared to their peers without internalizing psychopathology.

Lastly, it is important to consider developmental factors that may provide important context to this study’s findings. Youth with ADHD have been shown to have an approximately 33% delay in cortical thickness and EF (Berger et al., 2013). Considering the established links between EF and academic performance as well as moral reasoning (Cortés-Pascual et al., 2019; Vera-Estay et al., 2014), it is important to highlight that our sample may contain a broader range of development than expected given the relatively narrow age range. Another key consideration is that our sample of 10–12 year olds were recruited from numerous school districts with different building grade structures, meaning some of our participants attended school in a variety of different settings with varying academic demands and expectations (e.g., upper-elementary school, middle school, K-12 school). Together, the wide range in development and expectations associated with different academic settings could result in discrepancy between academic expectations and developmental ability. For example, a 12-year-old could have significant developmental delays in EF but attend a high school with increased academic demands compared to a 10-year-old with higher levels of EF in an elementary school. Thus, our participants may represent a broad range of development and be subjected to variable academic demands which may interact to inform perspectives on fairness. Future observational and intervention research should aim to explore how perceptions of fairness interact with increasing academic demands as early adolescents transition through academic settings.

### Implications

The stronger association between higher perceived school climate fairness and lower learning problems among adolescents with ADHD than adolescents without ADHD highlights a potential role of perceived fairness in understanding and managing academic functioning among these early adolescents. These results extend prior work by suggesting that fairness is not only a general feature of positive school climate, but may be particularly salient for youth who frequently encounter disciplinary action and experience challenges in navigating educational systems. By situating fairness within the broader context of ADHD and academic functioning, this study contributes to a more nuanced understanding of how relational and ecological factors shape outcomes for this population.

From an applied perspective, these findings are consistent with calls to foreground fairness and justice in educational policy and practice. Killen and Rutland (2022) have argued that promoting fairness is essential for supporting students’ well-being, and prior reviews have shown that schoolwide interventions can shift school climate (inclusive of perceptions of fairness; Charlton et al., 2021). For example, Mitchell and Bradshaw (2013) highlighted the potential of reducing the use of exclusionary discipline strategies (e.g., being sent to the principal’s office, suspension) in favor of proactive management strategies (e.g., establishing clear rules and expectations, using global and specific praise) to improve overall perceptions school climate. Although the present study does not test these practices directly, it suggests that interventions designed to enhance equitable climate may be particularly impactful for adolescents with ADHD.

Rather than positioning fairness as a direct target for individualized intervention, these findings suggest that fairness perceptions should be considered within broader models of school climate and academic functioning. Improved fairness perceptions may foster stronger engagement and belonging, which are in turn associated with positive academic outcomes (Chory, 2023; Wong, 2022). Future research should examine whether fairness operates as a mechanism through which schoolwide practices influence outcomes for adolescents with ADHD.

### Strengths, Limitations, and Future Directions

The present study contained several strengths, including a large sample, a multi-informant approach, and the use of well-validated measures to assess attitudes to perceived fairness, academic functioning, and ADHD status. However, it is also important to consider the limitations of the present study. First, given the cross-sectional nature of this study, we were not able to explore the directionality of the observed associations. Future research would benefit from examining the interactions of perceptions of fairness and academic functioning using longitudinal data to examine associations over time.

Several considerations regarding our measures are also important to note. We based our ADHD (and comparison) diagnosis on the K-SADS administered to the child’s caregiver. Given teacher-report data was unavailable for ~ 25% of the sample and teachers often observe students on medication when ADHD behaviors are likely to be reduced, we did not consider teacher ratings in making diagnostic determinations; rather, we assessed cross-setting symptoms and impairment were assessed solely through the K-SADS semi-structured interview. In addition, no objective measures of the participant’s school environment were available. Thus, it is unclear whether students’ lower perceptions of fairness were due to actual experiences of injustice or unfairness in the school setting, or by the misinterpretation of school environments that are not, in fact, unfair or unjust. Future studies should assess the differential role of the objective climate of a school compared to perceptions of the climate in academic functioning. One such way could be using discipline records or observational ratings of teachers as objective measures of fairness in coordination with rating scales, in line with prior research (Skiba et al., 2002; Wang & Degol, 2016). This study also did not distinguish between how fairly students felt they were treated personally versus how fairly they believed students at their school were treated overall. The questions in the measure used in this study ask the reporter to rate how “students at my school” are treated, rather than how they themselves feel that they are treated (Haynes et al., 1994). It is possible that an individual’s perception of how fairly they are treated is more closely linked than their perception of fairness at the school level to their academic functioning. Considering individuals with ADHD face significant stigmatization in general (Becker et al., 2017; Mueller et al., 2012), including from teachers (Metzger & Hamilton, 2021), examining the way they feel they are treated in schools is an important area for future research in this area. Additionally, objective measures of academic performance such as grades, homework completion records, or effort scores on report cards, could also be used to deepen our understanding of the types of academic domains that are related to perceptions of fairness. Relatedly, future research could use objective information via geocoding or gathering school district information (e.g., funding, access to educational resources) to contextualize the relation between perceptions of fairness and academic functioning with other environmental influences.

Finally, although not the focus of the current study, race and income were found to be significant predictors in the regression models for study skills and learning problems, respectively. In line with established research in these areas that highlights a variety of pathways in which constructs tied to race and economic status (e.g., access to less funded schools as a consequence of redlining, less financial resources to attain educational tools like computers or tutoring) (Lukes & Cleveland, 2021; Liu et al., 2022) may lead to poorer school performance, our results highlight two specific areas that may be related to these sociodemographic factors as found in our sample and important to consider further in future research. For example, a cluster analysis of factors including demographic and socioeconomic factors in addition to co-occurring conditions may facilitate further understanding of patterns among these variables and their associations or differences in perceptions of fairness. In turn, this might allow us to better predict which individuals might be particularly at risk of having poor perceptions of fairness depending on relevant demographic factors.

## Conclusion

This study contributes to our understanding of the role of school climate for adolescents with and without ADHD. To our knowledge, this is the first study to provide support for the association between the perceived fairness of the school climate and academic functioning among adolescents with ADHD. The results warrant further exploration into the role of perceived fairness, as well as other aspects of school climate, in the academic functioning of adolescents with ADHD. Further, it may be useful to target perceptions of school climate through intervention to improve academic outcomes for adolescents with ADHD. Future research should aim to test the efficacy of improving students’ attitudes to fairness on improving school functioning.

## Data Availability

The data are not publicly available but are available from the corresponding author upon reasonable request and after execution of a data use agreement.

## Footnotes

1 ADHD status and medication use were, as expected, strongly correlated (see Table 2). The pattern of findings was unchanged when medication use was not included as a covariate.
